# Differences in Angiographic Profile and Immediate Outcome of Primary Percutaneous Coronary Intervention in Otherwise Risk-Free Young Male Smokers

**DOI:** 10.7759/cureus.8799

**Published:** 2020-06-24

**Authors:** Salik Ahmed, Sanam Khowaja, Saher Khowaja, Tariq Ashraf, Kanwal Aamir, Mahesh K Batra, Musa Karim, Muhammad Anis M Ahmedani, Syed Z Jamal

**Affiliations:** 1 Adult Cardiology, National Institute of Cardiovascular Diseases, Karachi, PAK; 2 Internal Medicine, Aga Khan University Hospital, Karachi, PAK; 3 Cardiology, National Institute of Cardiovascular Diseases, Karachi, PAK; 4 Statistics, National Institute of Cardiovascular Diseases, Karachi, PAK; 5 Electrophysiology, National Institute of Cardiovascular Diseases, Karachi, PAK

**Keywords:** smoking, st-elevation myocardial infarction, young, angiography, primary percutaneous coronary intervention

## Abstract

Introduction

Cigarette smoking is a well-established risk factor for the development and progression of coronary artery disease (CAD) and it is strongly related to cardiac morbidity and mortality. Therefore, this study aimed to compare the angiographic profile and immediate clinical outcomes in young male smokers and non-smokers without any other cardiac risk factors presented with ST-elevation myocardial infarction (STEMI).

Methods

This study includes young (≤40 years) male patients presented without cardiac risk factors other than smoking. Angiographic profile and immediate outcome of primary percutaneous coronary intervention (PCI) were collected from the hospital database.

Results

A total of 580 young male patients were included in this study, 51.2% (297) were smokers. Baseline characteristics and presentation were similar for smoker and non-smoker groups. Angiographic profile was not significantly different for smokers in terms of pre-procedure thrombolysis in myocardial infarction (TIMI) flow (p = 0.373), the number of vessels involved (p = 0.813), infarct-related artery (p = 0.834), and left ventricular dysfunction (p = 0.311). Similarly, in-hospital outcomes of primary PCI were not significantly different in smokers. Post-procedure no-reflow was in 3.4% vs. 2.8%; p = 0.708, acute stent thrombosis in 1.7% vs. 0.4%; p = 0.114 and in-hospital mortality in 1.0% vs. 1.4%; p = 0.657 of the smoker and non-smoker group, respectively.

Conclusion

Our study concludes smoking has no significant impact on the angiographic profile and immediate clinical outcomes of primary PCI after STEMI in young males, without any other conventional cardiac risk factors. With these findings, further multicenter prospective studies are needed to identify other potential causes in such patients.

## Introduction

Asians are more than half of the world population and more than a quarter of the population of the developing world live in South Asian countries, namely, Pakistan, Bangladesh, India, Sri Lanka, Nepal, and Bhutan [1,2]. South Asian populations are known to have a higher tendency of coronary artery disease (CAD) and possess different genetic profiles and lifestyle than the western population [2]. Effective preventive measures for CAD in this population is crucial to curtailing the global disease burden. Identification and management of risk factors for CADs such as hypertension, hypercholesterolemia, diabetes, and smoking have proved to be effective preventive strategies in the western population [3]. In the last two decades, a transformation in dietary habits and lifestyle has been witnessed for the south Asian population due to urbanization and globalization of dietary behavior [3-6]. Such rapid transformation has led to an escalation of CAD in the region.

According to the World Health Organization (WHO), global annual deaths attributable to cardiovascular diseases (CVD) are around 17.9 million, and more than 75% of these cases are from developing countries [7]. CADs are considered to be the disease of old age, unfortunately, the incidence of CAD is increased among the younger population [8,9]. A local study reported cigarette smoking as the second most prevalent CVD risk factor, after increased BMI, among young (<45 years) patients [10]. A paradoxical phenomenon of favorable outcomes after acute myocardial infarction (AMI) was observed for smokers. In later studies, this phenomenon was attributed to the young age and lesser severe disease among smokers [11,12]. Smokers were found to develop ST-elevation myocardial infarction (STEMI) about 10 years earlier than non-smokers and smokers have higher sex- and age-adjusted one-year mortality [13].

Cigarette smoking is a well-established risk factor for the development and progression of coronary heart disease and is strongly related to morbidity and mortality and is a leading risk factor of CAD in our young population [10,13]. Data are scarce for the Pakistani population; therefore, this study aimed to compare the angiographic profile and immediate clinical outcomes in young male smokers and non-smokers without any other cardiac risk factor presented with STEMI and who underwent coronary intervention at a cardiac center in Karachi, Pakistan.

## Materials and methods

**Figure 1:**
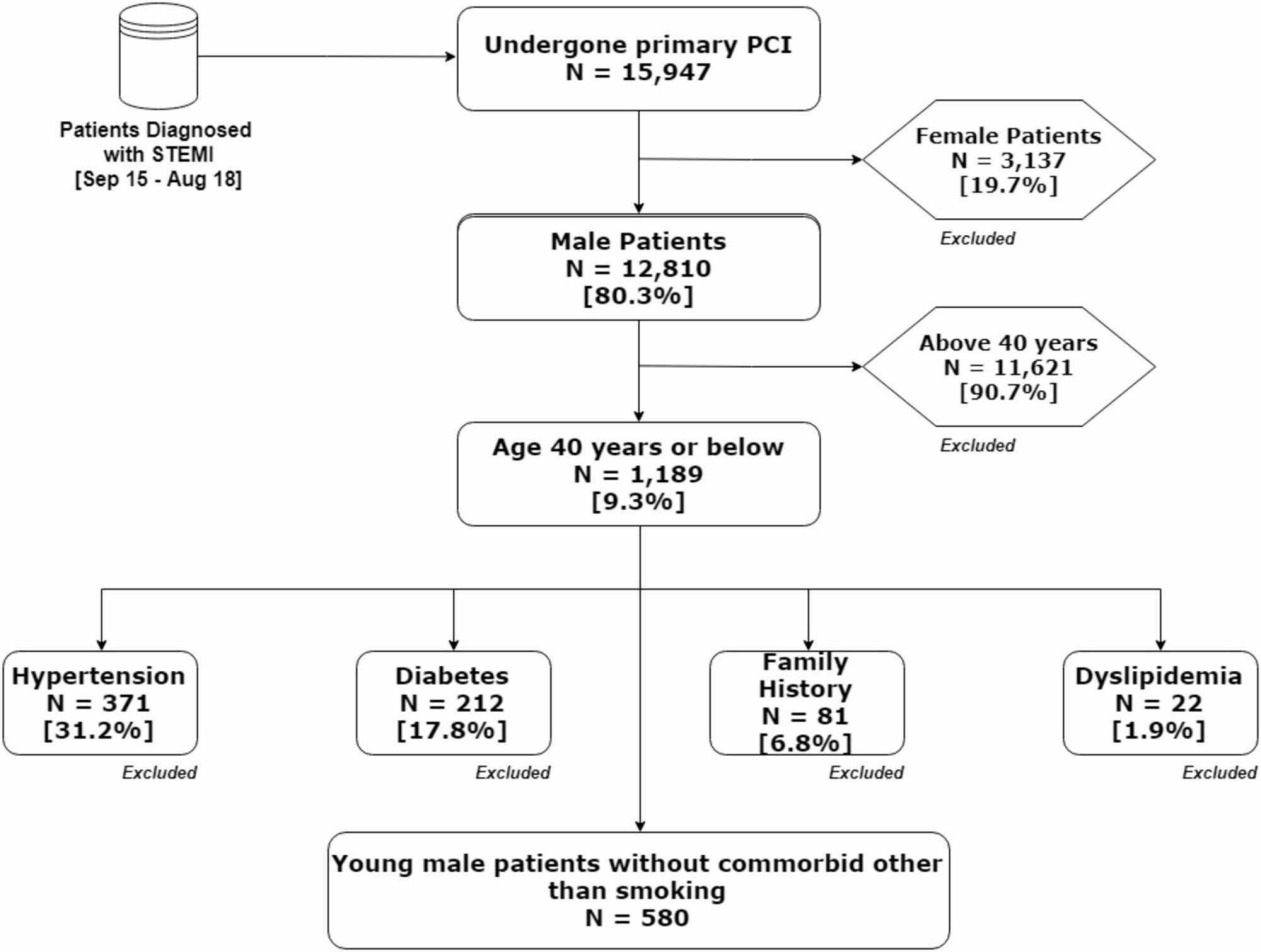
Patients selection criteria for the study PCI, percutaneous coronary intervention; STEMI, ST-elevation myocardial infarction

This retrospective observational study was conducted at the National Institute of Cardiovascular Diseases (NICVD) Karachi, Pakistan. The ethical review committee of the institute approved the study (ERC-33/2018) and in accordance with the Declaration of Helsinki, written informed consent was obtained from all patients regarding the procedure as well as the use of data for research purposes. Data for the study were extracted from the institutional primary percutaneous coronary intervention (PCI) database for the period from September 2015 to August 2018. As an institutional practice, demographics, presentation, diagnostic tests, and outcomes data were collected on pre-defined proforma, after patients consent, and anonymized data were available after institutional approval. In this study, we included young (<= 40 years) male patients who presented with STEMI and who undergone primary PCI. Patients with comorbid known risk factors of CAD, other than smoking, such as hypertension (HTN), diabetes mellitus (DM), family history of CAD, and dyslipidemia were excluded from the study. The patient selection criteria for the study were presented in Figure 1.

The STEMI was defined and managed as per the ACCF/AHA STEMI diagnosis and management guidelines. As a routine assessment, angiography and primary PCI procedures were performed and interpreted by consultant cardiologists with the experience of five years or more. After routine workup, electrocardiographic (ECG) and cardiac enzyme assessment, clinical presentation (KILLIP class), symptom onset to hospital arrival time, and door to balloon time were recorded. Patients history regarding known risk factors was obtained such as HTN (at least six months history of any antihypertensive medication), DM (at least six months history of any antihyperglycemic medication or insulin or glycated hemoglobin (HbA1c) > 6.5%), dyslipidemia (low-density lipoprotein cholesterol (LDL-C) levels > 130 mg/dL and/or high-density lipoprotein cholesterol (HDL-C) levels < 40 mg/dL), and family history (patient positive history of premature ischemic heart disease (IHD) in first degree relatives, male less than 55 years of age or female less than 65 years of age). Patients with a history of currently smoking five cigarettes a day for at least one year or equivalent were classified as smokers.

The angiographic profile of the patients was assessed in terms of chronic total occlusion (CTO), defined as thrombolysis in myocardial infarction (TIMI) flow grade 0, the number of diseased vessels, infarct-related (culprit) artery, lesion complexity, involvement of side branch, left ventricular (LV) dysfunction, and thrombus grading. Intra and post-procedure, in-hospital, outcomes include procedure success (TIMI flow grade III), peri-procedural use of intra-aortic balloon pump (IABP) and export catheter, no-reflow (TIMI flow grade 0-I), acute stent thrombosis, and all-cause in-hospital mortality.

Patients were divided into two groups, smokers and non-smokers. Demographic profile, angiographic profile and immediate outcomes of primary PCI were compared in two groups. Statistical analyses of the extracted data were performed using IBM SPSS Statistics for Windows, Version 21.0 (IBM Corp., Armonk, NY). Mean (± standard deviation) and percentage (frequency) were computed appropriately and the Mann-Whitney U test, for continuous variables, and chi-square test, for categorical variables, were performed. P ≤ 0.05 was taken as the level of significance.

## Results

A total of 580 young male patients were included in this study, 51.2% (297) were smokers. Smoker and non-smoker groups were similar in terms of age (35.29 ± 4.59 years vs. 35.54 ± 4.49 years, p = 0.575), symptom onset to hospital arrival time (238.87 ± 230.00 minutes vs. 239.19 ± 187.83 minutes, p = 0.145), and door to balloon time (80.35 ± 49.30 minutes vs. 83.78 ± 71.6 minutes, p = 0.622). Comparison of baseline characteristics of smokers and non-smokers are presented in Table 1.

**Table 1:** Comparison of baseline characteristics of smokers and non-smokers

Characteristics	Total	Smokers	Non-smokers	p-value
Total	580	297 (51.2%)	283 (48.8%)	-
Age (years)	35.41 ± 4.54	35.29 ± 4.59	35.54 ± 4.49	0.575
Body Mass Index (kg/m^2^)	25.79 ± 3.13	25.88 ± 3.58	25.71 ± 2.56	0.78
KILLIP class				
I	533 (91.9%)	272 (91.6%)	261 (92.2%)	0.612
II	24 (4.1%)	15 (5.1%)	9 (3.2%)	
III	11 (1.9%)	5 (1.7%)	6 (2.1%)	
IV	12 (2.1%)	5 (1.7%)	7 (2.5%)	
Symptom onset to hospital arrival time				
Mean ± SD minutes	239.18 ± 210.3	238.87 ± 230	239.5 ± 187.83	0.145
≤ 120 minutes	202 (34.8%)	110 (37%)	92 (32.5%)	0.253
> 120 minutes	378 (65.2%)	187 (63%)	191 (67.5%)	
Door to balloon time				
Mean ± SD minutes	82.02 ± 61.18	80.35 ± 49.3	83.78 ± 71.6	0.622
≤ 90 minutes	429 (74%)	221 (74.4%)	208 (73.5%)	0.802
> 90 minutes	151 (26%)	76 (25.6%)	75 (26.5%)	

Angiographic profile was not significantly different for smokers in terms of pre-procedural TIMI flow (p = 0.373), number of vessels involved (p = 0.813), infarct-related artery (p =0.834), and LV dysfunction (p = 0.311). A comparison of the angiographic profile of smokers and non-smokers are presented in Table 2.

**Table 2:** Comparison of the angiographic profile of smokers and non-smokers

Characteristics	Total	Smokers	Non-smokers	p-value
Total	580	297 (51.2%)	283 (48.8%)	-
Chronic total occlusion	287 (50.7%)	156 (54%)	131 (47.3%)	0.078
Number of vessels involved				
None	1 (0.2%)	1 (0.3%)	0 (0%)	0.813
Single vessel (SVD)	406 (70%)	206 (69.4%)	200 (70.7%)	
Two vessels (2VD)	119 (20.5%)	61 (20.5%)	58 (20.5%)	
Three vessels (3VD)	48 (8.3%)	25 (8.4%)	23 (8.1%)	
Left Main (LM)	6 (1%)	4 (1.3%)	2 (0.7%)	
Infarct related artery				
None	1 (0.2%)	1 (0.3%)	0 (0%)	0.834
Left anterior descending (LAD)	411 (70.9%)	211 (71%)	200 (70.7%)	
Right coronary (RCA)	104 (17.9%)	56 (18.9%)	48 (17%)	
Left circumflex (LCX)	52 (9%)	23 (7.7%)	29 (10.2%)	
Ramus	3 (0.5%)	1 (0.3%)	2 (0.7%)	
Left main (LM)	4 (0.7%)	2 (0.7%)	2 (0.7%)	
Diagonal	5 (0.9%)	3 (1%)	2 (0.7%)	
Lesion complexity				
None	4 (0.7%)	1 (0.3%)	3 (1.1%)	0.595
A	18 (3.1%)	12 (4%)	6 (2.1%)	
B	116 (20%)	59 (19.9%)	57 (20.1%)	
C	356 (61.4%)	178 (59.9%)	178 (62.9%)	
High C	51 (8.8%)	29 (9.8%)	22 (7.8%)	
Non high C	35 (6%)	18 (6.1%)	17 (6%)	
Side branch involvement	68 (11.7%)	40 (13.5%)	28 (9.9%)	0.181
Left ventricular dysfunction	328 (56.6%)	174 (58.6%)	154 (54.4%)	0.311
Thrombus grading				
No	43 (7.4%)	18 (6.1%)	25 (8.8%)	0.209
Possible	42 (7.2%)	19 (6.4%)	23 (8.1%)	
Small	61 (10.5%)	37 (12.5%)	24 (8.5%)	
Moderate	109 (18.8%)	52 (17.5%)	57 (20.1%)	
Large	85 (14.7%)	39 (13.1%)	46 (16.3%)	
Total	240 (41.4%)	132 (44.4%)	108 (38.2%)	

Similarly, immediate clinical outcomes of primary PCI were not significantly different in smokers. Post-procedure no-reflow was observed in 3.4% (10) vs. 2.8% (eight); p = 0.708, acute stent thrombosis in 1.7% (five) vs. 0.4% (one); p = 0.114 and in-hospital mortality in 1.0% (three) vs. 1.4% (four); p = 0.657 of the smoker and non-smoker group, respectively.

## Discussion

The CAD in the young patient has altogether different manifestations due to the differences in clinical outcomes, presentation, and risk profile. CAD is no longer remains the old age disease, it is becoming more prevalent among the young population due to increased risk profile such as smoking, diabetes, and sedentary lifestyles [8,14]. In clinical practice, premature STEMI comprises of 2%-12% of the total STEMI [8,14,15].

Among the other potential risk factors, such as unhealthy eating habits, obesity, and lack of physical activeness, smoking is the most prevalent risk factor [13]. A study conducted in our local population by Nadeem et al. [10] reported 46% current smokers among young (<45 years) patients with a history of IHD. Various other studies in the past reported smoking as high as more than 80% in patients with premature myocardial infarction (MI) [16,17]. In our study, we found 51.2% smokers among young (≤ 40 years) male patients presented with STEMI having no other cardiac risk factors such as diabetes, hypertension, dyslipidemia, and obesity.

Nicotine, carbon monoxide, and oxidant gases are the three basic constituents through which smoking deteriorates the cardiovascular system. The sympathomimetic effects of nicotine induce coronary artery vasoconstriction, leads to an increase in blood pressure, heart rate, and myocardial contractility [14,18]. Due to comparative more avid binding of carbon monoxide with hemoglobin, as compared to oxygen, it reduces the transportation of oxygen in the blood resulting in scarcity of oxygen within the heart [14,19,20]. Finally, the oxidant gases induce inflammation, cause endothelial damage, and increase plasma fibrinogen resulting in enhance coagulability. It also contributes to the thrombogenesis and platelet activation via oxidizing the LDL particles [19-21].

The smoker’s paradox, an observational paradoxical phenomenon of favorable outcomes after AMI in smokers, is a common observation of various studies; however, there exists a controversy regarding smoker’s paradox in young patients. Chen et al. concluded evidence to support the existence of a smoker’s paradox in young (≤ 45 years) patients, while, Liu et al. reported no paradoxical phenomenon in outcomes of AMI in young smokers [9, 22]. It is important to address this phenomenon because it not only a clinical decision dilemma for clinicians, but also it is misleading for smoker patients [22]. In our study, we observed no significant differences in in-hospital outcomes of smokers and non-smokers patients, with no other conventional cardiac risk factor, suggesting no evidence of smoker’s paradox in our young population.

To the best of our knowledge, this is the first study comparing the angiographic profile of young smokers and non-smokers, without any conventional cardiac risk factors, in our population. We found that male smokers and non-smokers had a similar clinical and angiographic profile in terms of KILLIP class at presentation, symptom onset to hospital arrival time, the extent of CAD, culprit vessel, lesion complexity, left ventricular function, and thrombus grading. Similarly, in-hospital outcomes such as post-procedure no-reflow, acute stent thrombosis, and mortality were similar for smoker and non-smoker sub-groups. Our study findings are aligned with the finding of a systematic review and meta-analysis conducted by Liu et al. [9]. But persistent smoking after revascularization is an independent predictor of long-term reoccurrence of major adverse cardiac events (MACE) [13,14]. Therefore, emphasis on smoking cessation has a pivotal role in reducing the risk of MACE after AMI. In literature, the rate of smoking cessation after AMI varies from 28% to 77% depending on the definition of quitting and smoking [23-25]. In-hospital, as well as post-discharge smoking cessation counseling and programs should be part of every cardiac rehabilitation program. As quitting smoking is a complex and difficult process; therefore, effective interventions, both pharmacological and psychological, are the need of the time.

Our study has several limitations, the study was a retrospective study; therefore, the impact of quantum of smoking (number of packs or sticks) and the duration of smoking on outcomes could not be assessed. Secondly, due to the small sample size and single-center coverage, the finding of insignificant differences among smokers and non-smokers needs further confirmation in multicenter prospective studies with a larger sample size.

## Conclusions

Our study concludes smoking has no significant impact on the angiographic profile and immediate clinical outcomes of primary PCI after STEMI in young males, without any other conventional cardiac risk factors. With these findings, further multicenter prospective studies are needed to identify other potential causes in such patients.
